# *In vitro* maturation is slowed in prepubertal lamb oocytes: ultrastructural evidences

**DOI:** 10.1186/1477-7827-12-115

**Published:** 2014-11-24

**Authors:** Maria G Palmerini, Stefania A Nottola, Giovanni G Leoni, Sara Succu, Xhejni Borshi, Fiammetta Berlinguer, Salvatore Naitana, Yerbol Bekmukhambetov, Guido Macchiarelli

**Affiliations:** Department of Life, Health and Environmental Sciences, University of L’Aquila, L’Aquila, Italy; Department of Anatomy, Histology, Forensic Medicine and Orthopaedics, La Sapienza University, Rome, Italy; Department of Veterinary Medicine, University of Sassari, Sassari, Italy; Catholic University “Our Lady of Good Counsel” Faculty of Pharmacy, Tirana, Albania; Marat Ospanov West Kazakhstan-State Medical University, Aktobe, Kazakhstan

**Keywords:** Immature oocyte, Cumulus-oocyte complexes, In vitro maturation, Ultrastructure, Lamb, Sheep

## Abstract

**Background:**

*In vitro* maturation (IVM) of immature oocytes retrieved from unstimulated ovaries may avoid side effects connected to hyperstimulation during IVF procedures, including the risk of cancer recurrence. In humans, the scarce availability of immature oocytes limits morphological studies. The monovular ovine may represent an experimental model for IVM studies.

**Methods:**

To assess if the scarce developmental competence of prepubertal oocytes (PO) is related to morphological changes we analyzed, by light and transmission electron microscopy, cumulus-oocyte-complexes (COCs) from lambs (30-40 days old) and sheep (4-6 years old) at sampling and after 7 h, 19 h, 24 h of IVM. Meiotic progression was determined at the same time points.

**Results:**

At sampling, the germinal vesicle (GV) of PO was round and centrally or slightly eccentrically located, whereas in adult oocytes (AO) it was irregularly shaped and flattened against the oolemma. PO, differently from AO, showed numerous trans-zonal projections. Organelles, including cortical granules (CGs), were more abundant in AO. After 7 h, the percentage of AO that underwent GVBD-MI transition increased significantly. In PO, the oolemma was juxtaposed to the ZP; in AO, it showed several spikes in correspondence of cumulus cells (CC) endings. In PO, organelles and isolated CGs were scattered in the ooplasm. In AO, groups of CGs were also present under the oolemma. After 19 h, PO underwent GVBD-MI transition; their oolemma showed several spikes, with CC projections retracted and detached from the ZP. AO underwent MI-MII transition; their oolemma regained a round shape. CGs were located beneath the plasmalemma, arranged in multiple, continuous layers, sometime discontinuous in PO. After 24 h, both groups reached the MII-stage, characterized by a regular oolemma and by expanded CCs. PO showed CGs distributed discontinuously beneath the oolemma, while AO showed a continuous monolayer of CGs.

**Conclusions:**

Even if PO were able of reaching morphological maturation after 24 h of IVM, our ultrastructural analysis allowed detecting the presumptive sequence of cytoplasmic alterations connected with the delay of nuclear maturation, that might explain the reduced developmental competence of such oocytes. Data from the sheep model are of interest for zootechny, and provide an experimental basis for improving human IVM technology.

## Background

*In vitro* maturation (IVM), followed by *in vitro* fertilization (IVF) and embryo transfer (ET) may restore fertility in humans, even in combination with cryopreservation [1, 2]. IVM may be useful as rescue measure in conventional IVF protocols by maturing retrieved GV-stage oocytes *in vitro* (*rescue IVM*). IVF cycles often make available a mixed cohort of MII, metaphase I (MI), and post mature, including immature germinal vesicle (GV) oocytes. The effective competence of these retrieved immature oocytes is under debate [3]. In IVM, maturation rates rarely exceed the 50-55% [2]. Defective cytoplasmic maturation seems to be at the basis of the low maturation rates of IVM oocytes [4].

IVM is applied with high yields in animal husbandry, not only to improve gamete preservation and to increase reproductive performance, but also to preserve endangered species or those of zootechnic interest [5, 6].

Controlled ovarian stimulation, IVM and oocyte/embryo cryopreservation are not feasible for young cancer patients, where hormonal ovarian stimulation could increase the risk of cancer recurrence [7]. In this case, the following strategies can be applied: i) *in vitro* growth (IVG) and IVF of oocytes from cryopreserved cortical biopsies, mainly containing primordial follicles [1, 8] or ii) the use of immature oocytes, cryopreserved at either the immature GV- or the mature MII-stage, i.e. before or after *in vitro* maturation (IVM) [9]. A chance for fertility preservation in prepubertals girls candidate to oncotherapy may be a pre-oncotreatment ovarian cortical strip, followed by cryopreservation and then, after disease recovery, a programmed IVG of thawed follicles, followed by IVM and IVF-ET of GV-stage oocytes [1].

Several ultrastructural and clinical studies demonstrated that a morphological assessment is necessary for evaluating the outcome of these procedures on the oocyte quality [10–14]. The scarce availability of human oocytes, especially in young patients, obviously limits morphological studies.

Indeed, animal models may be necessary to investigate the developmental competence of immature GV-stage oocytes subjected to IVM (to obtain MII-stage oocytes), and then to IVF. The ovine, a monovular species like human, could potentially represent an optimal animal model, being closer to human reproductive physiology than other species [15]. However, ovine prepubertals oocytes (PO) may show a poor development competence that can be likely also accounted for a fine morphological impairment [16, 17].

Therefore, we aimed to study by light and transmission electron microscopy the morphology of COCs obtained from prepubertal lambs before, during and at the end of IVM, in order to describe the timing of nuclear and cytoplasmic maturation, to evidence eventual alterations in respect to adult sheep, and ultimately to determine COCs quality. In this report, we account ultrastructural evidence of a prepubertal oocyte IVM impairment.

## Methods

All chemicals in this study were obtained from Sigma Chemical CO. (St. Louis, MO, USA) unless stated otherwise.

Ovaries of slaughtered *Sarda* breed lambs (30-40 days old) and sheep (4-6 years old) were transported to the laboratory within 1-2 hours using *Dulbecco Phosphate Buffered Saline* solution (PBS) and antibiotics. Follicles with a diameter higher than 2 mm were sliced with a microblade and their content released in medium TCM199 (with Earle’s salts and bicarbonate) supplemented with 25 mmol HEPES, 0.1 g/L penicillin, 0.1 g/L streptomycin and 0.1% (w/v) polyvinylalcohol (PVA). COCs with 4-10 layers of granulosa cells, oocyte with uniform cytoplasm, homogenous distribution of lipid droplets and outer diameter of about 90 μm, were selected. Samples were washed 3 times in the same fresh medium and matured *in vitro* in TCM 199 supplemented with 10% heat-treated oestrus sheep serum (OSS), 1 IU/mL of FSH/LH and 100 μM cysteamine. 40-45 COCs were put in 500 μL of maturation medium in four-well culture dishes (Nunclon, Nalge Nunc International, Denmark), covered with 300 μL of mineral oil and cultured for 24 h in 5% CO2 in air at 39°C. *In vitro* maturation (IVM) experiments were performed at least 3 times.

### Analysis of meiotic progression during IVM

IVM was performed as above described. At each time point (0, 7, 19 and 24 hours of *in vitro* culture), oocytes (n. 40 per each experimental groups) were decumulated by gentle pipetting using a narrow bore glass capillary, fixed in ice cold methanol, and incubated with 10 μg/mL Hoechst 33342 in ice-cold methanol for 15 min.

Stained oocytes were mounted into a small droplet of glycerol on a glass slide and examined under an epifluorescence inverted microscope (Nikon Diaphot, Japan).

### Morphological evaluation by light and transmission electron microscopy (LM and TEM)

Prepubertal and adult COCs were fixed at retrieval (0 hour) and at different intervals during IVM (7, 19 and 24 hours) and then processed for LM and TEM. Per each experimental group were used 15 prepubertal and 20 adult COCs, obtained from at least three different animals. Methods of LM and TEM preparative were adapted from those previously described [18, 19]. Briefly, COCs were fixed in 1.5% in Glutaraldehyde (SIC, Roma, Italia) in PBS solution for at least 2-5 days at 4°C. Samples were rinsed three times for about 10 minutes in PBS, post-fixed with 1% osmium tetroxide (Agar Scientific, Stansted, UK) in PBS and rinsed again in PBS. COCs were then embedded in small blocks of 1% of agar of about 5x5x1 mm in size and dehydrated in ascending series of ethanol (Carlo Erba Reagenti, Milano, Italia). Samples were immersed in propylene oxide (BDH Italia, Milano, Italia) for solvent substitution, embedded in epoxy resin (Electron Microscopy Sciences, Hatfield, PA, USA) and sectioned by a Reichert-Jung ultracut ultramicrotome. Semithin sections, of 1 μm thick, were stained with toluidine blue, examined by light microscopy (Zeiss Axioskop) and photographed by digital camera (Leica DFC 230). Ultrathin sections (60-80 nm) were cut by a diamond knife, mounted on copper grid and contrasted with saturated with Uranyl Acetate and Lead Citrate (Sic Roma, Italia). Finally, COCs were examined and photographed using Zeiss EM 10 and Philips MET CM 100 Electron Microscopes operating at 80 KV.

For the evaluation by LM and TEM, the following parameters were taken into consideration: general features (e.g. shape and dimension) of the oocyte and cumulus cells, shape and location of the nucleus, type and quality of organelles, integrity of the oolemma and the zona pellucida (ZP), appearance of the perivitelline space (PVS) (width, presence of fragments) [18, 19].

### Statistical analysis

Differences in the maturation progression were subjected to the Chi squared analysis. Statistical analysis was performed using the statistical software program Statgraphic Centurion XV (version15.2.06 for Windows; StatPoint, Inc., Herndon, VA, USA) and a probability of P ≤0.05 was considered the minimum level of significance.

## Results

### Analysis of meiotic progression during IVM

At sampling (IVM: 0 hours) all the oocytes were arrested at GV-stage in lambs and adults. After 7 hours of IVM, the GV break-down (GVBD) occurred in the 45.7% of PO and 39.6% of adult oocytes (AO). MI rates were significantly higher in AO than in PO (34.7% *vs.* 14.1%, P < 0.01). Similarly, at 19 hours of IVM, AO (70.4%) reached MII-stage earlier than PO (36.3%) (P < 0.001). PO showed MII-stage rates comparable with AO only after 24 hours of IVM (95.1% *vs.* 96.7%), with the remaining oocytes arrested at GV-stage (Table 1).

**Table 1:** **Percentages of meiotic progression in prepubertal and adult ovine cumulus-oocytes-complexes (COCs) at sampling (IVM: 0 hours) and after 7, 19 and 24 hours of IVM**

	Prepubertal Oocytes (PO)	Adult Oocytes (AO)
***IVM: 0 hrs***	GV: 100%	GV: 100%
***IVM: 7 hrs***	GV: 40.2%^(a)^	GV: 25.7%^(b)^
	GVBD: 45.7%	GVBD: 39.6%
	MI: 14.1%^(a)^	MI: 34.7%^(b)^
***IVM: 19 hrs***	GV: 5.1%	GV: 3.2%
	MI: 58.5%^(a)^	MI: 26.5%^(b)^
	MII: 36.4%^(a)^	MII: 70.3%^(b)^
***IVM: 24 hrs***	GV:4.6%	GV: 3.3%
	MI: 0.3%	-
	MII: 95.1%	MII: 96.7%

Values in the same row with different superscripts are significantly different (P < 0.01).

### Morphological evaluation by LM and TEM

#### IVM: 0 hours

##### General appearance

**Figure 1:**
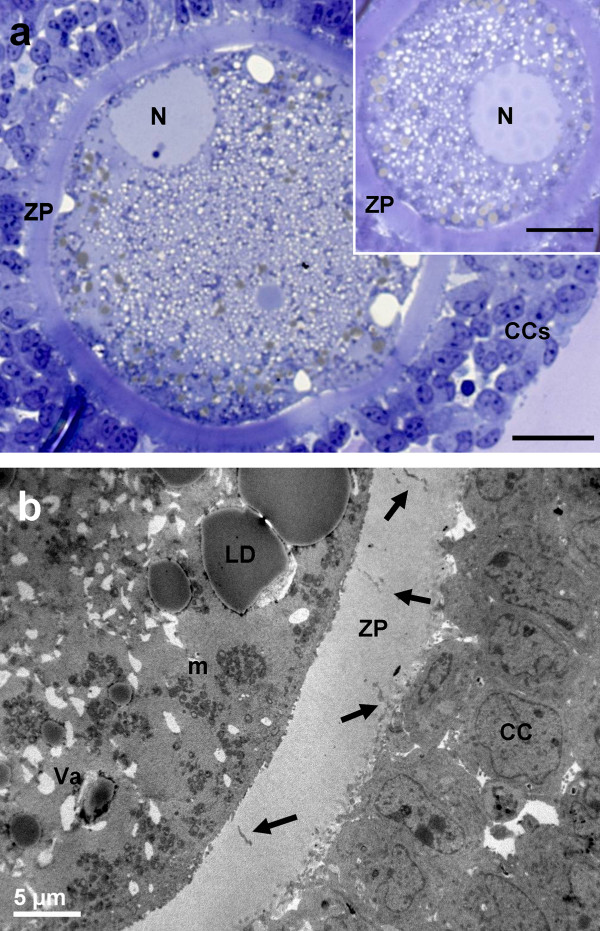
**General morphology and organelle microtopography in prepubertal ovine cumulus-oocyte-complexes (COCs) at sampling (IVM: 0 hours). a)** Representative LM image showing the eccentric or more central profile (*inset to Figure* 1 *a*) of the nucleus (N). Many layers of compact cumulus cells (CCs) adhere to a round zona pellucida (ZP). The ooplasm is rich of vacuoles (in white), lipid droplets (in grey) and organelles (in violet). Bar: 25 μm; inset bar: 20 μm. **b)** Representative TEM micrograph showing the oocyte and cumulus cells ultrastructure. Compact CCs establish connection to the oocytes by trans-zonal projection (arrows). Mitochondria (m), are interspersed to electron-negative vacuoles (Va), electron-dense lipid droplets (LD). Bar: 5 μm.

**Figure 2:**
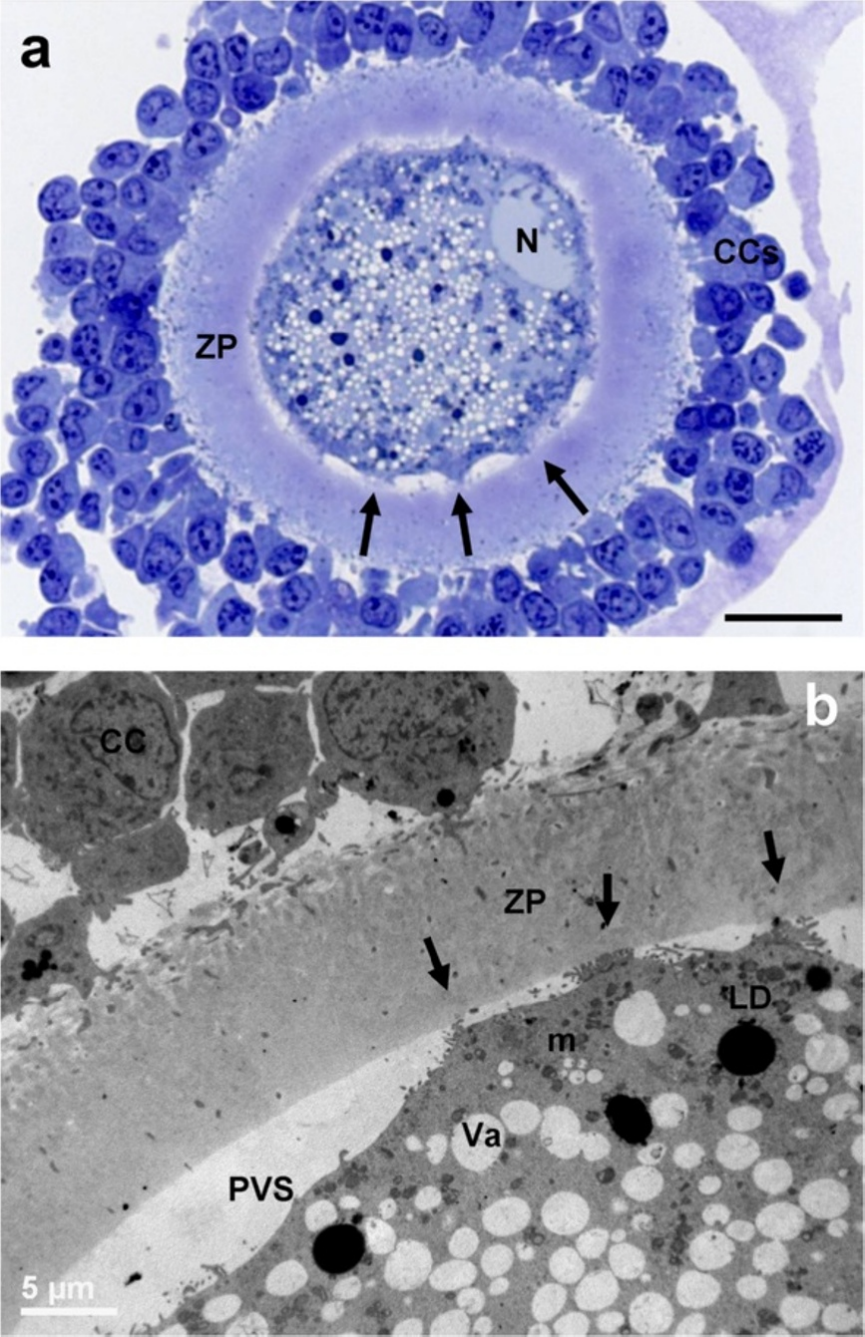
**General morphology and organelle microtopography in adult ovine cumulus-oocyte-complexes (COCs) at sampling (IVM: 0 hours). a)** Representative LM image showing the eccentric profile of an irregularly shaped nucleus (N), flattened against the ZP. Arrows indicate “spikes” of the oolemma. Many layers of compact CCs adhere to a round ZP. The ooplasm is rich of vacuoles (in white), lipid droplets (in grey) and organelles (in violet). Bar: 25 μm. **b)** Representative TEM micrograph showing the oocyte and CC ultrastructure. Cumulus expansion starts as indicated by the reduction of CC coupling. The oolemma is anchored to the ZP only in correspondence of focal adhesions (arrows) established with trans-zonal projections still present. ZP: zona pellucida; PVS: perivitelline space; CC: cumulus cell. Mitochondria (m), are interspersed to electron-negative vacuoles (Va), electron-dense lipid droplets (LD). Bar: 5 μm.

At sampling, both PO and AO showed numerous layers of compacted cumulus cells, a continuous ZP and a thin PVS. Ooplasms appeared rich of mitochondria, normal vacuoles and lipid droplets (Figures 1a, 2a).

##### Cumulus cell-oocyte interactions

In PO, the ZP showed numerous trans-zonal projections, connecting the oocyte with the surrounding cumulus cells (CCs) (Figure 1b), and the oolemma was rounded and regularly juxtaposed to the ZP. In AO, the oolemma appeared not uniformly connected to CCs, except than in correspondence of some focal adhesions where trans-zonal projections were still present (Figure 2b). This could be accounted for the onset of retraction and detachment of cumulus cell projections, providing a sort of traction of the oolemma toward the ZP giving a characteristic pointed shape (spikes) (Figure 2b).

##### Nucleus

The oocytes presented a round or oval GV bounded by a continuous nuclear envelope. In PO, the GV was located in a central or slightly eccentric ooplasmic position (Figure 1a and *inset*). In AO, the nucleus was always eccentric and flattened under the plasma membrane (Figure 2a).

##### Organelle and cytoplasmic subdomain

**Figure 3:**
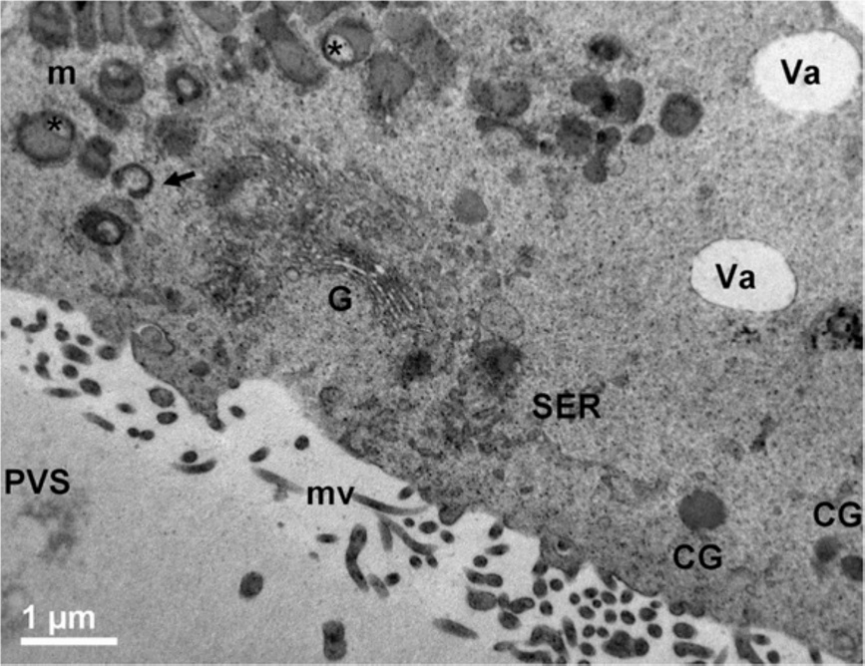
**Ultrastructure and organelle distribution in adult ovine COCs.** Representative TEM micrograph showing a well developed cup-shaped Golgi complex (G), in close proximity to the smooth endoplasmic reticulum (SER) and round, ovoid or hooded mitochondria (m). Irregularly shaped and electron-lucent vacuoles (Va) are delimited by a discontinuous membrane. Arrow: a hooded mitochondrion; asterisks: mitochondria with the so-called “clear vesicle” inside. CGs: round, electron-dense cortical granules; mv: microvilli; PVS: perivitelline space. Bar: 1 μm.

In all groups, vacuoles were often irregularly shaped, and bounded with a membrane, sometime discontinuous. These vacuoles were empty (Figure 3), except than for the occasional presence of cellular debris; they were often adjacent to electron-dense lipid droplets and to clusters of round-to-ovoid mitochondria. Well-developed SER, Golgi apparatus and electron-dense CGs were present in the ooplasm of AO (Figure 3). All the oocytes showed a continuous layer of microvilli.

#### IVM: 7 hours

##### General appearance

**Figure 4:**
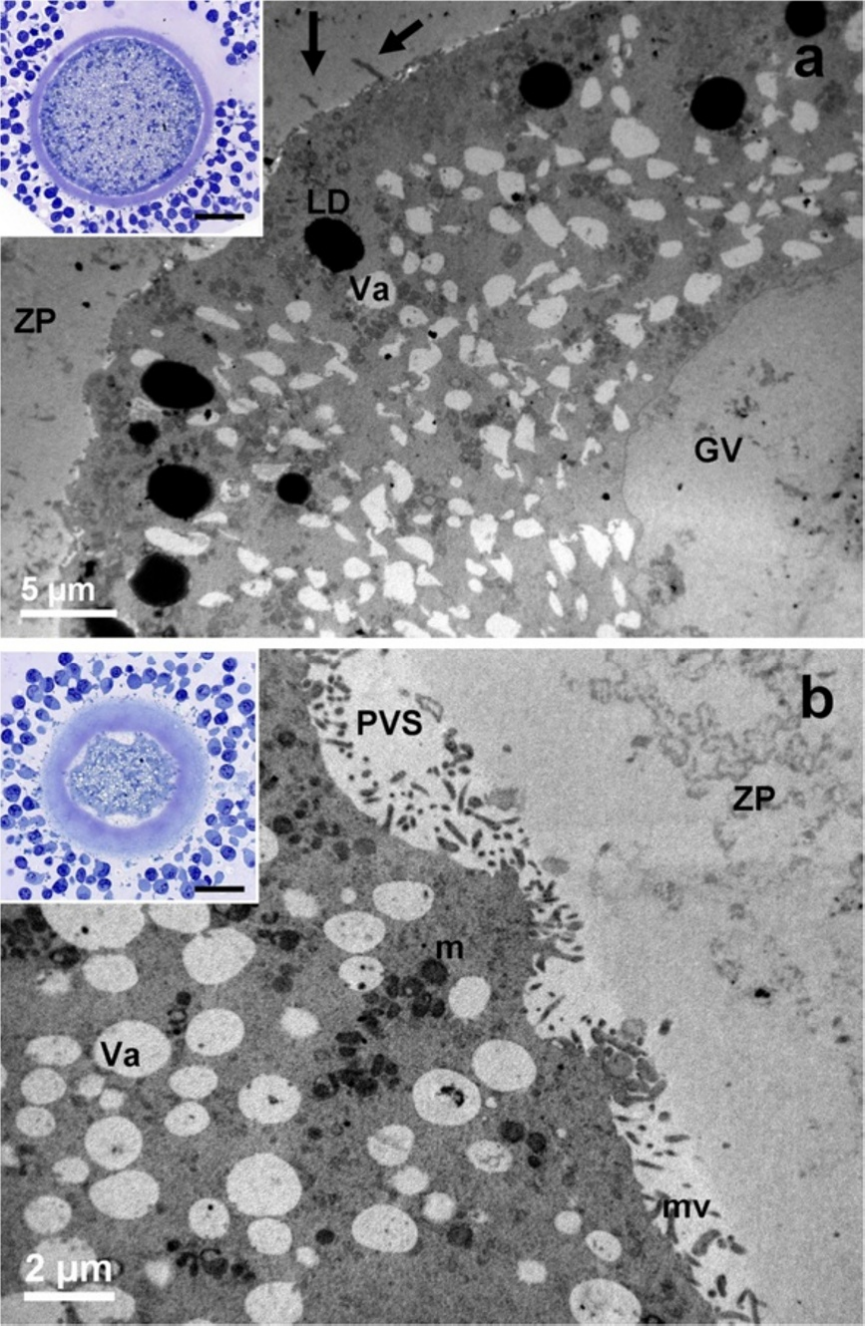
**General morphology and organelle microtopography in prepubertal (a) and adult (b) ovine cumulus-oocyte-complexes (COCs) after 7 hours of IVM. a)** Representative TEM micrograph showing the germinal vesicle (GV), the oolemma juxtaposed to the ZP and trans-zonal projections (arrows). Bar: 5 μm. *Inset in a)*: representative LM image showing the round shape of the oocyte. Bar: 20 μm. **b)** Representative TEM micrograph showing spikes of the oolemma. The nucleus is not present. A layer of microvilli (mv) is visible in the perivitelline space (PVS). Va: vacuole; m: mitochondria; LD: lipid droplets. Bar: 2 μm. *Inset in b)*: representative LM image showing spikes of the oolemma, uncontinuously adherent to the ZP. The apparent increased thickness of the zona pellucida, the wide PVS and scarce cytoplasm are effects of the section plane (not equatorial). Bar: 20 μm.

After 7 hours of IVM, LM observation did not reveal gross cytoplasm alterations (insets of Figure 4).

##### Cumulus cell-oocyte interactions

In AO, cumulus cell retractions were more abundant if compared to controls (Figure 2a and inset of Figure 4b, respectively). Only in AO, the oolemma showed spikes connected to the prolongation endings of retracted cumulus cells (Figure 4b).

##### Nucleus

**Figure 5:**
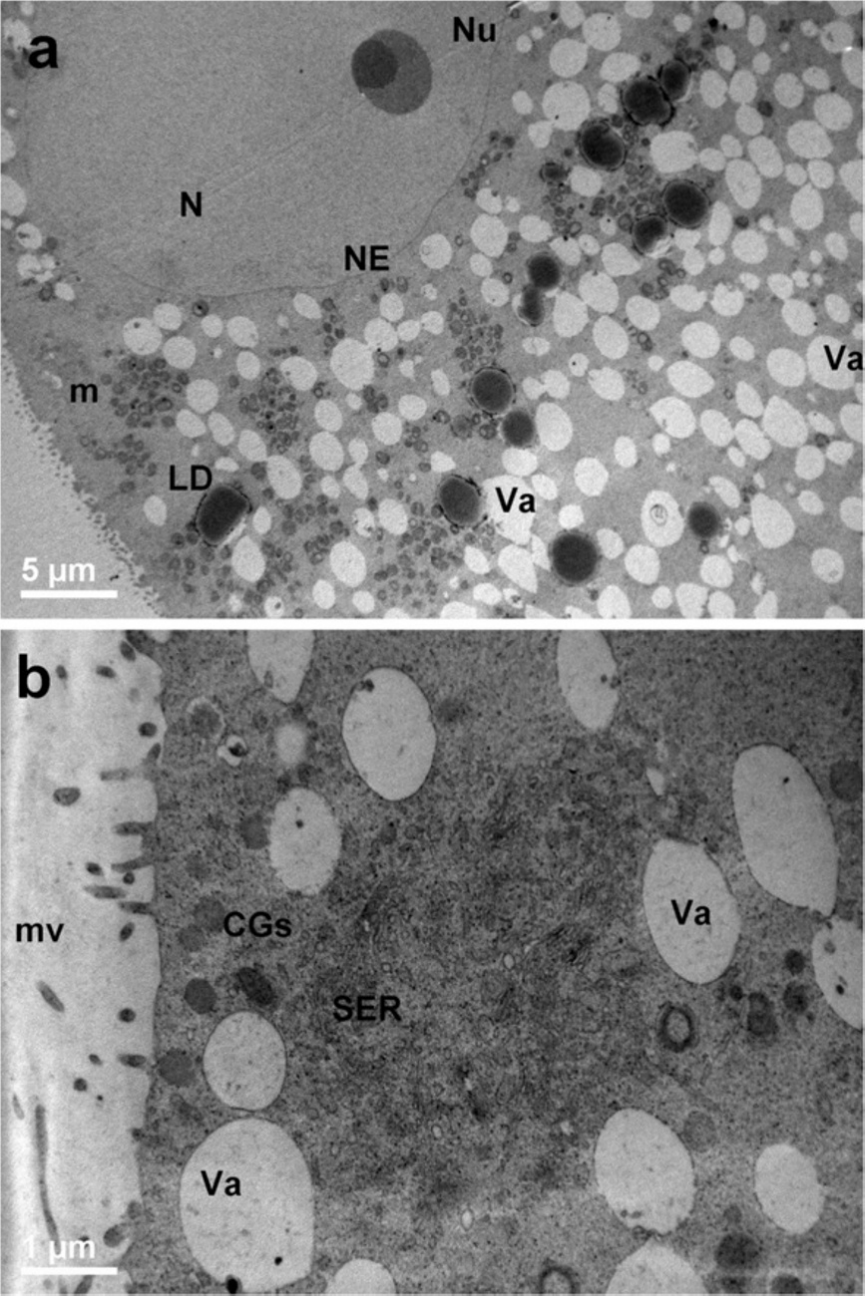
**Ultrastructure and organelle distribution in prepubertal (a) and adult (b) ovine cumulus-oocyte-complexes (COCs) after 7 hours of IVM. a)** Representative TEM micrograph showing the oocyte with no signs of nuclear reactivation. The nucleus (N) is surrounded by a complete and uniform nuclear envelope (NE) and a round electron-dense nucleolar body (Nu). Bar: 5 μm. **b)** Representative TEM micrograph showing an ooplasm with several vacuoles (Va) provided with an electron-dense membrane, and a SER close to electron-dense cortical granules (CGs); mv: microvilli. Bar: 1 μm.

In AO meiotic maturation resumed (Figures 4b, 5b), whereas in PO the nucleus persisted (Figure 4a), as shown by the presence of a continuous nuclear membrane, the chromatin dispersion and by a low number of round electron-dense nucleoli, consisting of tightly packed fibrillar material (Figure 5a).

##### Organelles and cytoplasmic subdomain

SER tubules and elements of the Golgi complex were present in both PO and AO. Numerous groups of CGs were present in the ooplasm of AO, even in subplasmalemmal regions (Figure 5b). In PO, isolated CGs were prevalent. In both PO and AO, a continuous microvillar layer projected from the oolemma surface into the PVS.

#### IVM: 19 hours

##### General appearance

**Figure 6:**
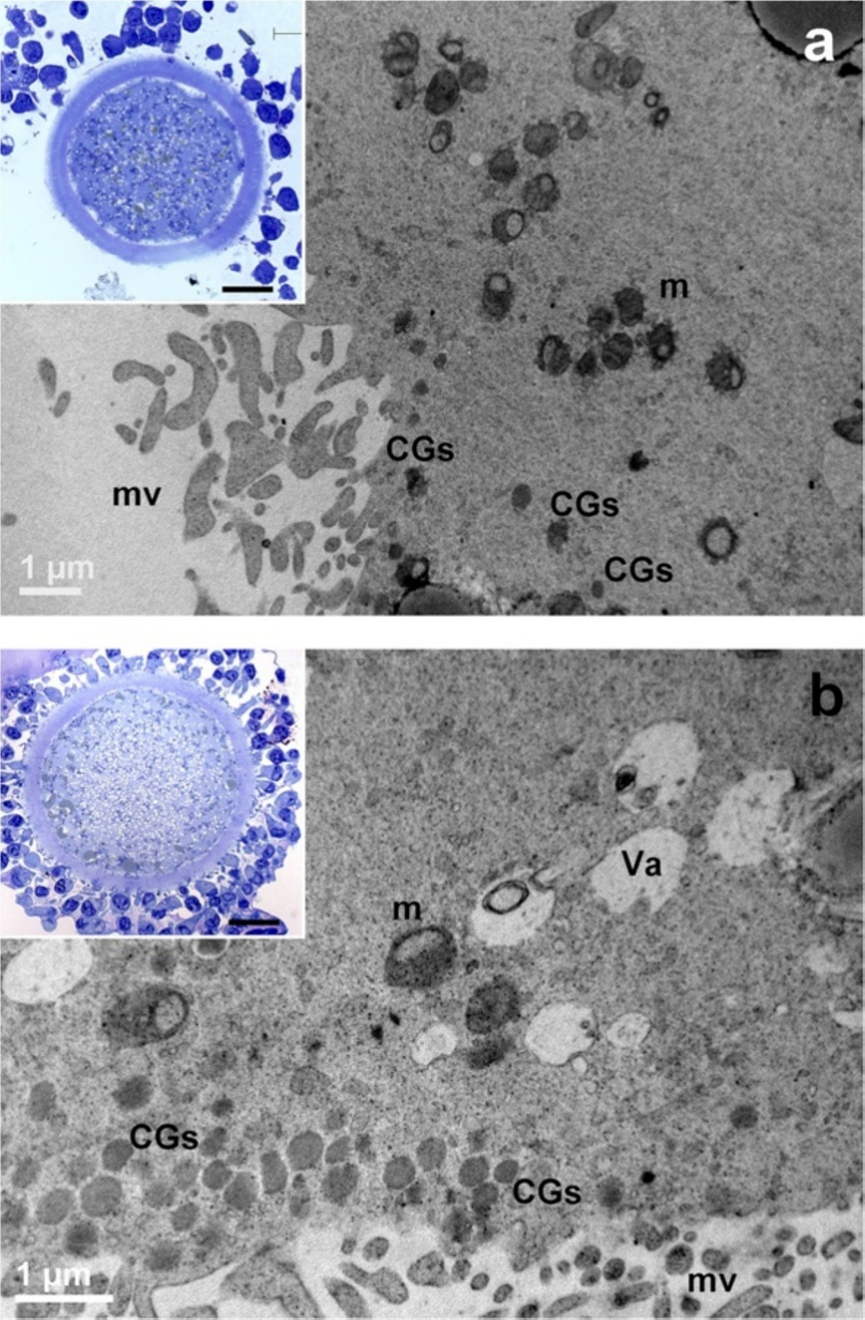
**General morphology and organelle microtopography in prepubertal (a) and adult (b) ovine cumulus-oocyte-complexes (COCs) after 19 hours of IVM. a)** Representative TEM micrograph showing an oocyte cortex with mitochondria (m), provided by numerous *cristae*. Cortical granules (CGs) are irregularly distributed in the sub-plasmalemmal region; mv: microvilli. Bar: 1 μm. *Inset in a)*: representative LM image showing spikes of the oolemma, associated with CC detachment. Bar: 20 μm. **b)** Representative TEM micrograph showing an ooplasm with several irregularly shaped vacuoles (Va), mitochondria (m) and multiple layers of electron-dense cortical granules (CGs). Bar: 1 μm. *Inset in b)*: representative LM image showing a regularly round shaped oolemma; mv: microvilli. Bar: 20 μm.

After 19 hours of IVM, cumulus expansion was visible in both groups by LM (insets to Figure 6).

##### Cumulus cell-oocyte interactions

Spikes of the oolemma appeared also in PO, associated with the retraction and detachment of cumulus cell projections, anchored to the ZP (inset to Figure 6a). AO presented a regularly round shaped oolemma, which seemed to restore its original aspect (inset of Figure 6b).

##### Nucleus

In PO, the absence of the GV and the expansion of cumulus cells characterized the meiotic resumption (Figure 6a).

##### Organelles and cytoplasmic subdomain

The ooplasm showed vacuoles and lipid droplets uniformly distributed. Multiple layers of CGs stratified in the sub-plasmalemmal area, but less uniformly in PO (Figure 6). CGs appeared round and electron-dense, in both PO and AO (Figure 6).

#### IVM: 24 hours

##### General appearance

At the end of IVM, PO and AO showed a spherical shape and a continuous PVS, surrounded by a compact ZP*.*

##### Cumulus cell-oocyte interactions

**Figure 7:**
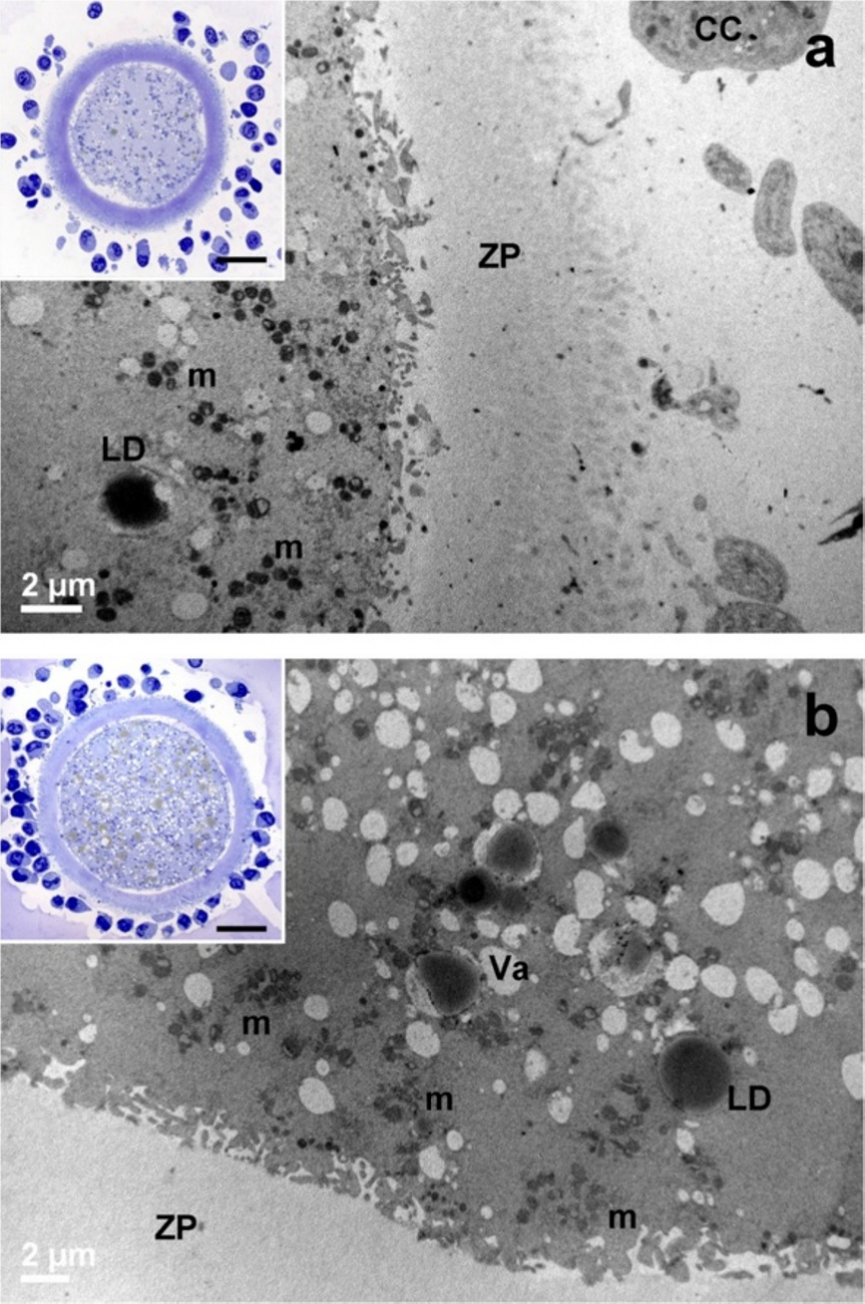
**General morphology and organelle microtopography in prepubertal (a) and adult (b) ovine cumulus-oocyte-complexes (COCs) after 24 hours of IVM. a)** Representative TEM micrograph showing expanded cumulus cells (CC), a zona pellucida (ZP) devoid of CC prolongations and a roundish oolemma. Bar: 2 μm. *Inset in a)*: representative LM image showing CC expansion after detachment from the ZP. Bar: 20 μm. **b)** Representative TEM micrograph showing several mitochondrial clusters (m), electron-lucent vacuoles (Va) and electron-dense lipid droplets (LD). Bar: 2 μm. *Inset in b)*: representative LM image of a round oocyte, provided with a continuous perivitelline space (PVS) and surrounded by a compact zona pellucida (ZP)*.* Bar: 20 μm.

In both PO and AO, most of CCs appeared expanded, even if a few of them remained still anchored to the ZP (insets of Figures 7, Figure 7a). The oolemma seemed to retain its physiological adhesion to the ZP, especially in AO (inset of Figure 7b).

##### Organelles and cytoplasmic subdomain

**Figure 8:**
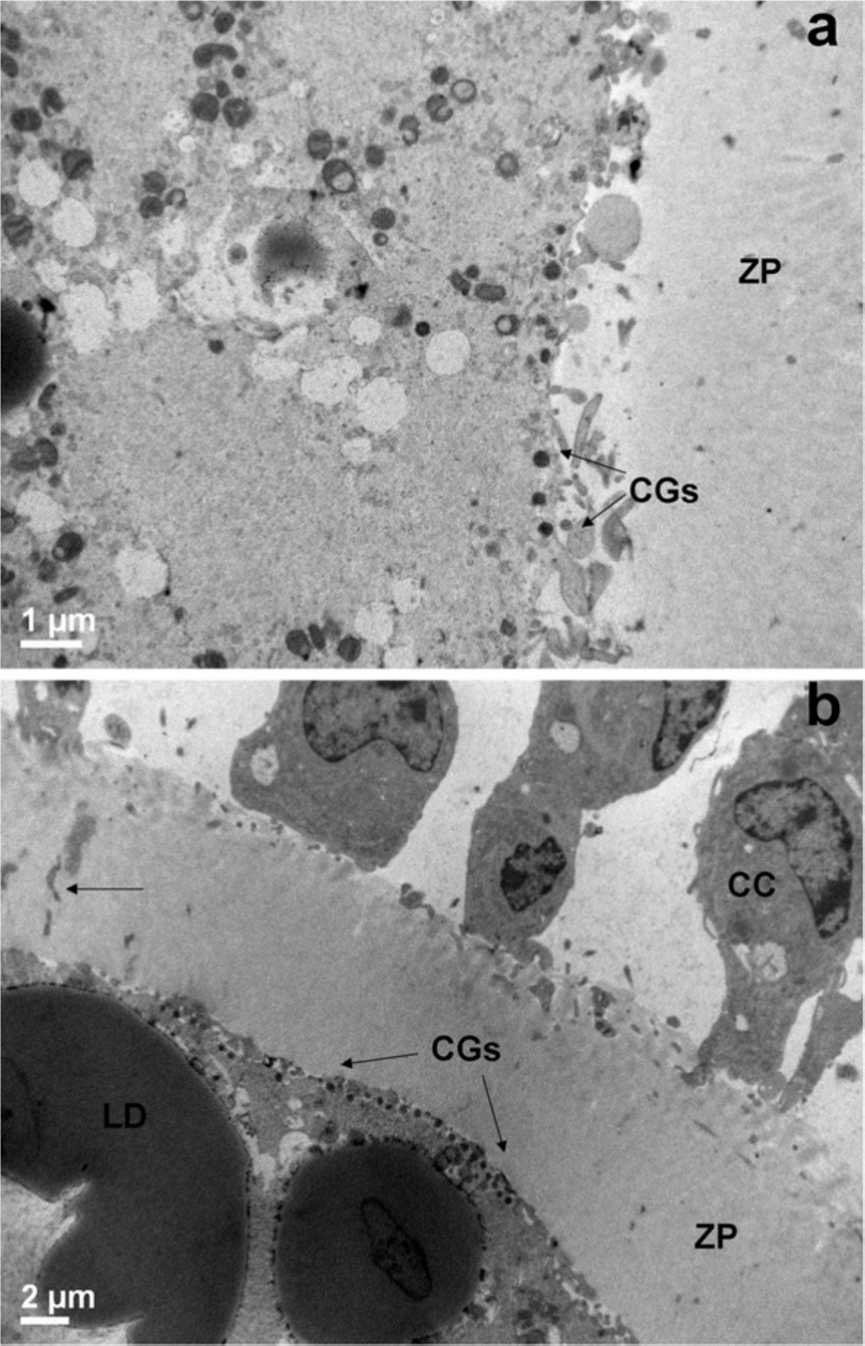
**Cortical granule distribution in prepubertal (a) and adult (b) ovine cumulus-oocyte-complexes (COCs) after 24 hours of IVM.** Representative TEM micrograph showing a discontinuous cortical granule (CG) distribution under the oolemma, with area devoid of CGs **(a)** and a continuous monolayer of CGs **(b)**. CC: a cumulus cell. Bar in **a)**: 1 μm. Bar in **b)**: 2 μm.

In all groups, the ooplasm presented numerous mitochondria clusters, clear vacuoles and high electron-dense lipid droplets (Figure 7). In PO, the distribution of CGs beneath the oolemma was discontinuous (Figure 8a). In AO, a continuous monolayer of CGs was present beneath the oolemma (Figure 8b). In all groups, microvilli covered the oolemmal surface.

##### Nucleus

Neither PB1 nor GV were detected in our samples.

## Discussion

In this study, we described the fine morphology of COCs obtained from prepubertal lambs and adult sheep during IVM in standard conditions.

The precise and differentiated time setting of sampling - from the beginning (0 hours) to the end (24 hours) of IVM - used for collecting prepubertal and adult ovine COCs, allowed us to obtain original data on the ultrastructural changes of the oocytes during whole culture period.

This sampling model was fundamental to detect morphological differences between prepubertals and adults. Specifically, the LM and TEM evaluation of meiotic progression evidenced in prepubertal animals a delay in the nuclear maturation and alterations in the cytoplasmic maturation. In fact, in PO the GVBD-MI transition occurred with 12 hours of delay respect to AO (7 hours of IVM vs. 19 hours, respectively). The occurrence of similar percentages of meiotic progression rates after 7 hours of IVM, in both GV- and GVBD-stages, may also account for this delay. Differently, at this timing, the majority of AO normally reached GVBD/MII stage. Indeed, the MI-MII transition was faster in AO than PO (most of the AO were at MII-stage after 19 hours of IVM) even if, as in AO, nuclear maturation ended in 24 hours also in PO, with the achievement of the MII-stage.

These findings are likely due to an alteration of the specific and coordinated sequence of events between the oocytes and surrounding CCs. In the immature oocyte, CCs tightly connect to each other and to the oocyte by means of intercellular cytoplasmic processes presenting junctional complexes, including gap junctions that facilitate exchange of nutrients, small signalling molecules and ions. Oocytes depend on the cumulus cells for metabolism of glucose and supply of pyruvate for energy production [20] and references herein cited]. Indeed, the GVBD depends on the uncoupling of CCs from the oocyte [20]. Thus, the stage of nuclear maturation well correlates with a different CC arrangement, as also clearly shown *in vitro* by our morphological data. Interestingly, in our data, the CC stage seemed to be associated to the nuclear phase, independently of the age of animals. When the nucleus was arrested at GV-stage, CCs were adherent to the ZP with numerous trans-zonal projections, responsible for the bi-directional communication between the oocyte and CCs. Differently, when meiosis resumed, CCs retracted by gradually detaching their trans-zonal projections from the ZP. This probably determined a tension on the oolemma that acquired the spiky shape. At MII-stage, when most of the CCs appeared already detached from the ZP, the oocyte had the recovery time necessary for restoring its original roundish shape beneath the ZP.

The heterocellular metabolic cooperation is so important to influence directly the bioenergetic requirements in the embryo after fertilization [21]. According to this, the delay we found in the nuclear maturation of PO seemed associated to alterations of the oolemma and contacts with somatic cells. The alterations of latter structures could be responsible for the poor developmental competence of PO.

The association between nuclear stages and morphological changes of the oolemma shape and CC distribution well correlates to the changes of shape and position of the nucleus, irregular and flattened against the oolemma in adult GV-stage oocytes or roundish and slightly eccentric in prepubertal GV-stage oocytes. The above, in agreement with what previously reported in sheep [22], could represent an ultrastructural parameter for the evaluation of the nuclear maturity, indicating the ability to resume meiosis *in vitro* and progress up to MII-stage.

*In vivo*, the follicle-oocyte dialogue synchronizes the activation of oocyte growth and its developmental capacity and release. This process is coordinated by bi-directional signals received and transmitted through ovarian somatic cells, in particular the CCs [20]. Oocytes enter a critical stage of development in response to diminishing cAMP (3’-5’-cyclic adenosine monophosphate) after the LH (luteinizing hormone) surge, resuming meiosis and progressing through a precisely synchronized nuclear and cytoplasmic maturation, to achieve full developmental competence [21].

*In vitro*, the physical removal of COCs from ovarian follicles results in spontaneous resumption of meiosis (largely because of a decrease in cAMP concentrations via phosphodiesterase type 3, PDE3), causing asynchrony between cytoplasmic and nuclear maturation and decreased oocyte developmental competence. In this view, innovative approaches to IVM are currently proposed in order to modulate *in vitro* cAMP concentrations within ovine COCs [23] or to inhibit PDE3 [24], to delay spontaneous nuclear maturation, and to improve developmental competence, with subsequent embryo viability.

Our ultrastructural analysis on prepubertal and adult ovine oocytes matured *in vitro* allowed not only to detect peculiar variations in PO, but also to individuate the timing of the delay in the nuclear maturation and the presumptive sequence of cytoplasmic alterations connected with it. Electron microscopy again resulted one of the techniques of choice for the evaluation of oocyte quality.

The ultrastructure of oocyte nucleus has been studied in lambs and sheep, mainly focusing on the nucleolus [25]. Other authors found that the animal size corresponded to a different oocyte nucleolar activity. While small sized lambs showed by TEM a vacuolated oocyte nucleolus, with a fibrillar center located at the nucleolar periphery, in adult sized lambs and sheep, vacuoles disappeared and the nucleolus showed an electron-dense fibrillar sphere with a fibrillar centre attached to it in the form of a halo [25]. Interestingly, our *in vitro* matured lamb oocytes showed a similar nucleolar morphology to that found in adult sized lambs and sheep *in vivo* [22, 25], thus indicating that IVM conditions could sustain a *quasi* physiological maturation.

Prepubertals showed modifications in the oocyte cytoplasmic maturation, mainly related to an altered CG distribution. In adult sheep oocytes, CGs relocate towards the ZP earlier than in prepubertal lamb during IVM, and finally aligned in a continuous monolayer under the oolemma, at MII stage. Prepubertal and adult goat oocytes subjected to IVM and IVF showed a similar CGs distribution [26]. Differences in CG size and volume fraction were also present *in vitro* in prepubertal and adult sheep oocytes [16].

Concerning the morphology of other organelles, our data are generally in agreement with previous studies on ovine oocyte ultrastructure. In fact, the morphology of mitochondria, lipid droplets, Golgi complexes and endoplasmic reticulum, before and after maturation, were comparable with previous reports on prepubertal and adult sheep oocytes matured *in vitro* [16, 17, 22] or *in vivo* [27–29]. As well, we observed that the organelle redistribution during maturation was coincident with what described by others, both *in vitro* and *in vivo* [16, 28].

The vacuole is a characteristic organelle that in the ovine oocyte is physiologically present in large numbers, differently from other species including humans. In humans, a large number of vacuoles appear only during degenerative process [14, 19]. In ovine, vacuoles are usually numerous and distributed around the GV [16, 28], even if they may be found even increased, in some pathological/experimental conditions [27]. In our samples, we did not found a significant variation of the vacuole amount. However, differently from what previously described, we did not found a dense assemblage of vacuoles in the center of the oocyte ooplasm [16, 27]. As well, we hardly found the localization of Golgi complexes around the GV [28]. These minor ultrastructural differences can be likely related to the different breeding investigated previously and the different experimental conditions.

## Conclusions

In conclusion, even if most PO achieved a nuclear and cytoplasmic maturation after 24 hour of IVM as the adult counterpart, the ultrastructural differences observed by LM and TEM during IVM, evidenced a delay in nuclear maturation and variations in the cytoplasmic maturation that might explain the reduced developmental competence of PO.

Since the interest in using prepubertal animals in zootechny, mainly related to the reduction of the generational interval and to the increase in the reproductive efficiency, is limited by an impaired developmental competence [16, 17, 30–34], our data could be useful to further optimize IVM conditions.

In young women, the comprehension of mechanisms regulating a correct nuclear and cytoplasmic maturation *in vitro*, from immature oocytes and in absence of ovarian stimulation [35], would means to offer a possibility to preserve fertility maintaining the ability for young cancer patients to have biological children [36].
